# Annexin-1 Mediates Microglial Activation and Migration via the CK2 Pathway during Oxygen–Glucose Deprivation/Reperfusion

**DOI:** 10.3390/ijms17101770

**Published:** 2016-10-22

**Authors:** Shuangxi Liu, Yan Gao, Xiaoli Yu, Baoming Zhao, Lu Liu, Yin Zhao, Zhenzhao Luo, Jing Shi

**Affiliations:** 1 Department of Neurobiology and Key Laboratory of Neurological Diseases of Hubei Province, Tongji Medical College, Huazhong University of Science and Technology, 13 Hangkong Road, Wuhan 430030, China; liushxi780815@sina.com (S.L.); xy_gaoxueyan@163.com (Y.G.); yuxiaoli123062@163.com (X.Y.); zb_ming@sina.com (B.Z.); Luliu8899@outlook.com (L.L.); zhaoyin85@hotmail.com (Y.Z.); lzzbvy@163.com (Z.L.); 2 Institute for Brain Research, Collaborative Innovation Center for Brain Science, Huazhong University of Science and Technology, 13 Hangkong Road, Wuhan 430030, China; 3 Medical College, Yichun Uiversity, 576 Xuefu Road, Yichun 336000, China; 4 Department of Center for Molecular Medicine, Medical College, Hubei University of Arts and Science, Xiangyang 441053, China

**Keywords:** ANXA1, microglia, activation, migration, OGD/R, CK2

## Abstract

Annexin-1 (ANXA1) has shown neuroprotective effects and microglia play significant roles during central nervous system injury, yet the underlying mechanisms remain unclear. This study sought to determine whether ANXA1 regulates microglial response to oxygen–glucose deprivation/reperfusion (OGD/R) treatment and to clarify the downstream molecular mechanism. In rat hippocampal slices, OGD/R treatment enhanced the ANXA1 expression in neuron, the formyl peptide receptor (FPRs) expression in microglia, and the microglial activation in the CA1 region (cornu ammonis 1). These effects were reversed by the FPRs antagonist Boc1. The cell membrane currents amplitude of BV-2 microglia (the microglial like cell-line) was increased when treated with Ac2-26, the N-terminal peptide of ANXA1. Ac2-26 treatment enhanced BV-2 microglial migration whereas Boc1 treatment inhibited the migration. In BV-2 microglia, both the expression of the CK2 target phosphorylated α-E-catenin and the binding of casein kinase II (CK2) with α-E-catenin were elevated by Ac2-26, these effects were counteracted by the CK2 inhibitor TBB and small interfering (si) RNA directed against transcripts of CK2 and FPRs. Moreover, both TBB and siRNA-mediated inhibition of CK2 blocked Ac2-26-mediated BV-2 microglia migration. Our findings indicate that ANXA1 promotes microglial activation and migration during OGD/R via FPRs, and CK2 target α-E-catenin phosphorylation is involved in this process.

## 1. Introduction

Microglia are the resident immune cells of the central nervous system (CNS) and function as critical regulators of the brain microenvironment; thus, these cells play significant roles after CNS injury [1,2]. They have been shown to migrate to areas of infarct where they are able to either protect mildly injured cells or induce cell death in severely damaged cells. Additionally, microglia clear cellular debris and toxic substances via phagocytosis [3,4]. Therefore, migration is an important functional property of microglial activation. Although many endogenous substances such as adenosine triphosphate (ATP) and cannabinoids are known to induce or regulate microglial migration [5,6,7], additional chemokines and regulators of microglial migration remain to be described.

Novel regulators of microglial migration can be found by assessing gene and protein expression changes during and after injury. Annexin-1 (ANXA1) is a glucocorticoid-regulated phospholipid-binding protein with both pro- and anti-inflammatory effects. Experiments on mouse had shown that ANXA1 plays a protective role in ischemia [8]. Approaches to protecting brain from cerebral injury, such as hydrogen sulfide inducing hypothermia and chloral hydrate preconditioning, significantly regulated ANXA1 expression in post-ischemic brain area [9,10]. Exploring the mechanism of ANXA1 playing a neuroprotective role is of great significance to preventing and prevent stroke. Oxygen–glucose deprivation/reperfusion (OGD/R)-induced microglial activation results in the synthesis and secretion of annexin-1 (ANXA1) [11]. Previous data have indicated that the expression of ANXA1 in the CNS is restricted mainly to microglia [12]. However, the pathological and physiological significance of ANXA1 expression in the CNS and its almost exclusive restriction to the microglia remains unclear [13]. In addition, the action of ANXA1 is mediated partly by activation of formyl peptide receptors (FPRs), a receptor family that comprises a group of seven trans-membrane G-protein-coupled receptors (GPCRs) [14]. Although roles for FPRs have been extensively characterized in neutrophils and monocytes, their functions in neurons and glia are poorly understood.

A new concept has emerged that posits a critical role for ANXA1 in phagocytosis [15]. There is also evidence that ANXA1 is a novel endogenous ligand that mediates apoptotic cell engulfment [16]. Recently, ANXA1 was reported to induce the migration of primary microglia in the resting state, and this effect was blocked by inhibiting FPRs with the antagonist Boc1 [17]. However, the effect of ANXA1 on microglial migration in response to OGD/R has not been reported, and the signaling mechanisms through which ANXA1 operates are not fully characterized.

We hypothesized that FPRs are among the receptors that alert microglia to neuronal injury. We therefore set out to determine the function of ANXA1/FPR signaling in microglial activation and migration in response to OGD/R as well as the underlying downstream molecular mechanisms. Using a combination of electrophysiology, migration assays, and pharmacologic manipulations, we determined that ANXA1 promotes microglial activation and migration via FPRs, α-E-catenin phosphorylation, and CK2 signaling.

## 2. Results

### 2.1. ANXA1 Expression in Hippocampal Slice Neurons Was Significantly Enhanced by OGD/R

**Figure 1:**
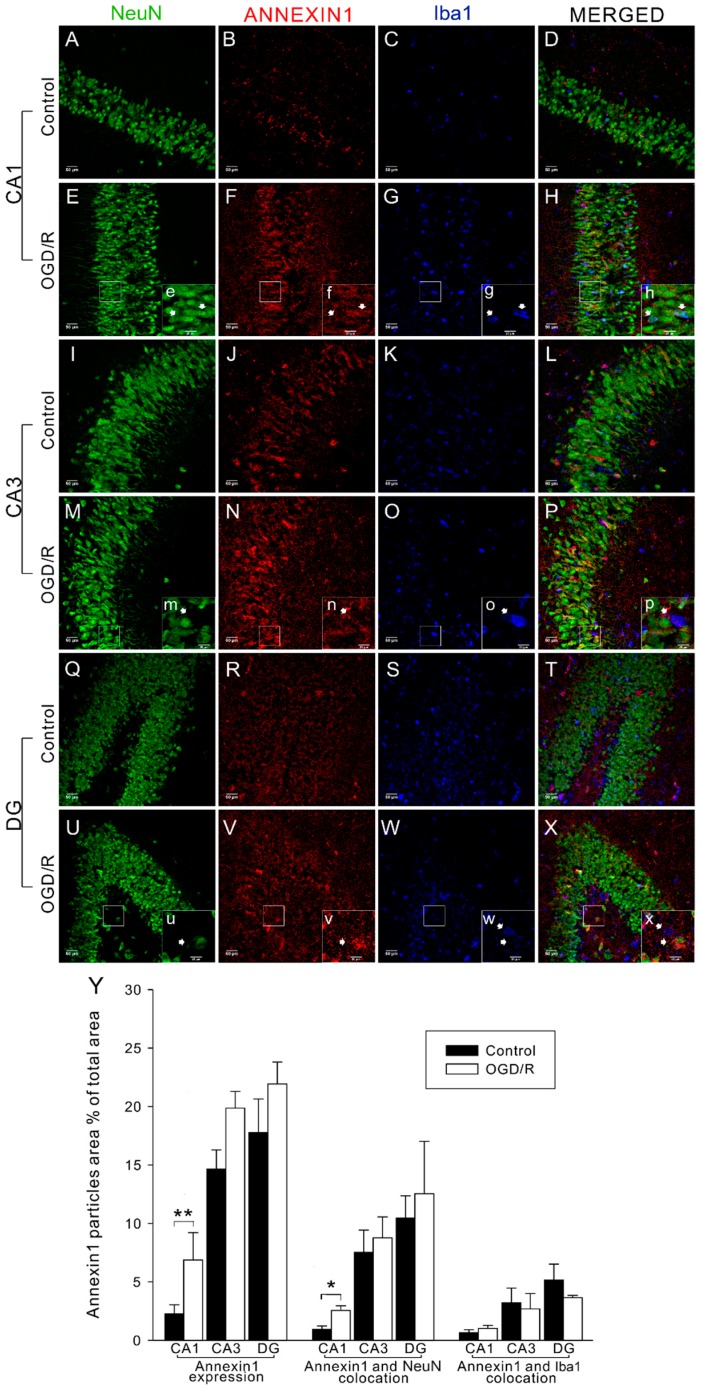
Immunofluorescence imaging revealed that annexin-1 (ANXA1) expression was increased by oxygen–glucose deprivation/reperfusion (OGD/R) in hippocampal slices. (**A**–**X**) Examples of neuron-specific nuclear protein (NeuN)-labeled neurons (**green**), ANXA1-positive particles (**red**), and ionized calcium binding adapter molecule 1 (Iba1)-labeled microglia (**blue**); in each picture, the right large area with lowercase letter is enlarged from the left small area in white box; the white arrows point at labeled cells and the corresponding area; scale bar: 50 μm; (**Y**) Fluorescence analysis revealed ANXA1 expression level and the level of ANXA1 co-localization with NeuN or Iba1 in CA1, CA3, and dentate gyrus (DG). The bars indicate the mean ± standard error of the mean (SEM) (*n* = 5–8). * *p* < 0.05, ** *p* < 0.01.

To determine the extent of ANXA1 expression, we carried out immunofluorescence imaging of organotypic hippocampal slice cultures (OHSCs). ANXA1-positive neurons were found throughout the cornu ammonis (CA1 and CA3) and dentate gyrus (DG) (Figure 1), a result that is consistent with enriched expression. After OGD/R, the number of ANXA1-positive cells was significantly increased compared with controls in the CA1 (Figure 1Y; *p* < 0.01). To confirm neuronal expression of ANXA1 and assess its expression pattern in microglia, we carried out ANXA1 co-labeling with NeuN (neuron-specific nuclear protein) and Iba1 (ionized calcium binding adapter molecule 1), respectively. In the CA1, CA3, and DG, ANXA1 was found to co-localize with both NeuN and Iba1 (Figure 1). In the CA3 and DG, the number of ANXA1-positive particles co-localized with NeuN was higher than the number of ANXA1-positive particles co-localized with Iba1 (Figure 1Y). Furthermore, the number of ANXA1-positive particles co-localized with NeuN was increased throughout the hippocampus after OGD/R treatment compared with controls, and this difference was significant in the CA1 (Figure 1Y; *p* < 0.05). In contrast, the number of ANXA1-positive particles co-localized with Iba1 was not increased by OGD/R treatment in any region (Figure 1Y; *p* > 0.05).

### 2.2. Microglial Expression of FPRs in Hippocampal Slices Was Significantly Enhanced by OGD/R

**Figure 2:**
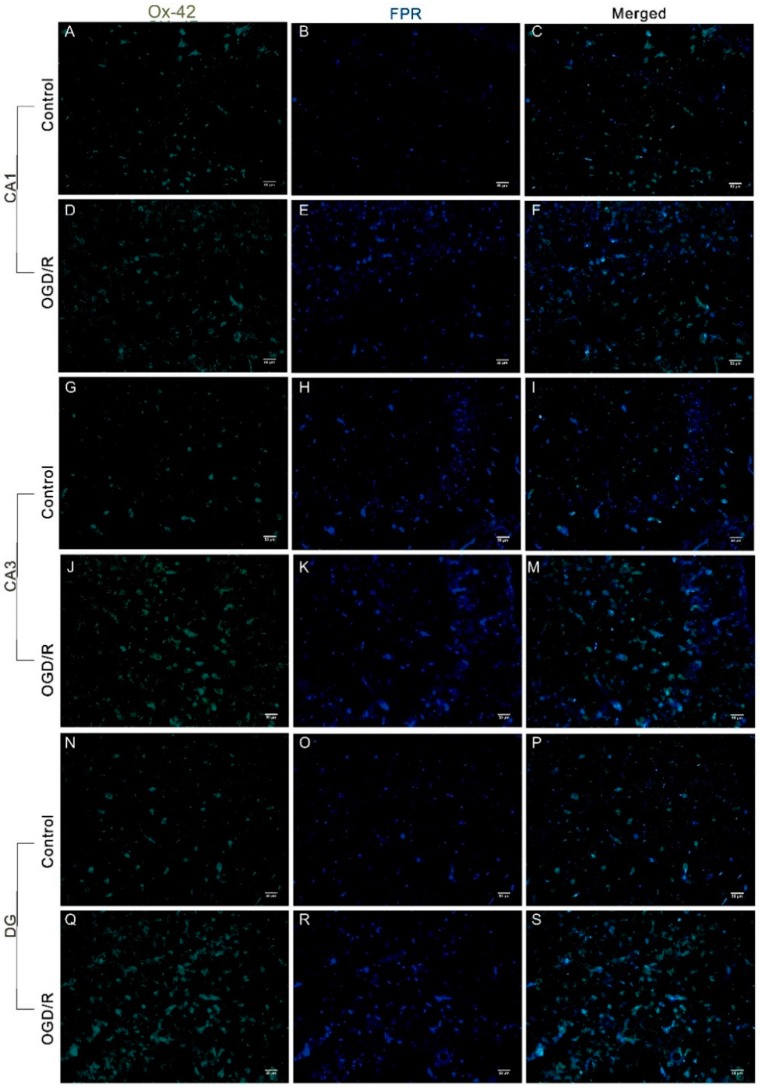

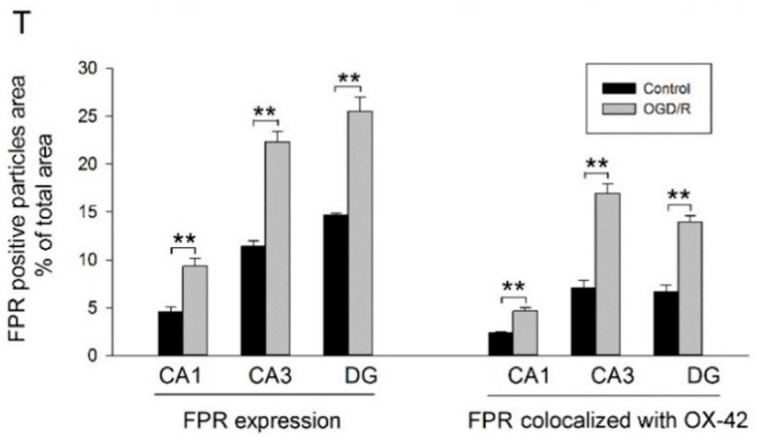
Oxygen–glucose deprivation/reperfusion (OGD/R) significantly elevated expression of formyl peptide receptors (FPRs) in hippocampal slices. (**A**–**S**) Examples of OX-42 (complement receptor Ш)-labeled microglia (**green**) and FPR-positive particles (**blue**); scale bar: 50 μm; (**T**) fluorescence analysis illustrated the expression level of FPRs and the levels of FPRs co-localized with OX-42 in the CA1, CA3, and dentate gyrus (DG) before and after OGD/R. The data are expressed as the mean ± standard error of the mean (SEM) (*n* = 3–8). ** *p* < 0.01.

**Figure 3:**
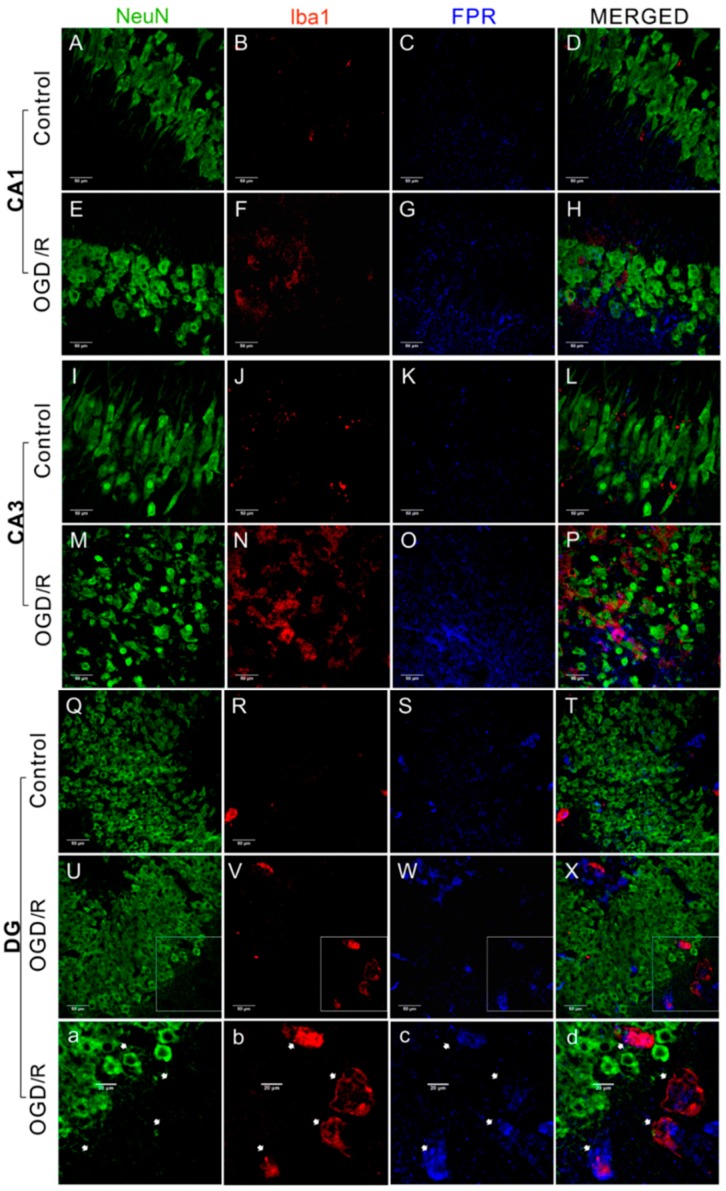
Immunofluorescence imaging revealed that formyl peptide receptors (FPRs) were rarely co-localized with neurons in hippocampal slices. (**A**–**X**,**a**–**d**) Examples of a neuron-specific nuclear protein (NeuN)-labeled neuron (**green**), Iba1-labeled microglia (**red**), and FPR-positive particles (**blue**). The white arrows point at labeled cells and the corresponding area. Scale bar: 50 μm.

We next assessed the expression of FPRs in OHSCs by immunofluorescence. FPR-positive particles were observed in the CA1, CA3, and DG (Figure 2 and Figure 3). Expression of FPRs was significantly enhanced by OGD/R treatment compared with controls in all tested regions (Figure 2T; *p* < 0.01). To assess cell-type specific expression, neurons were labeled with NeuN and microglia were labeled with Iba1 or OX-42 (complement receptor III. FPR-positive particles co-localized mostly with OX-42-positive microglia (45.5% to 76.0% co-expression depending on FPR) (Figure 2T). In contrast, FPR-positive particles co-localized with NeuN were rarely detected (Figure 3). In all three regions assessed, the number of FPR-positive particles co-localized with OX-42 significantly increased after OGD/R treatment compared with controls (Figure 2T; *p* < 0.01).

### 2.3. ANXA1 Acts via FPRs to Enhance Microglial Activation during OGD/R

**Figure 4:**
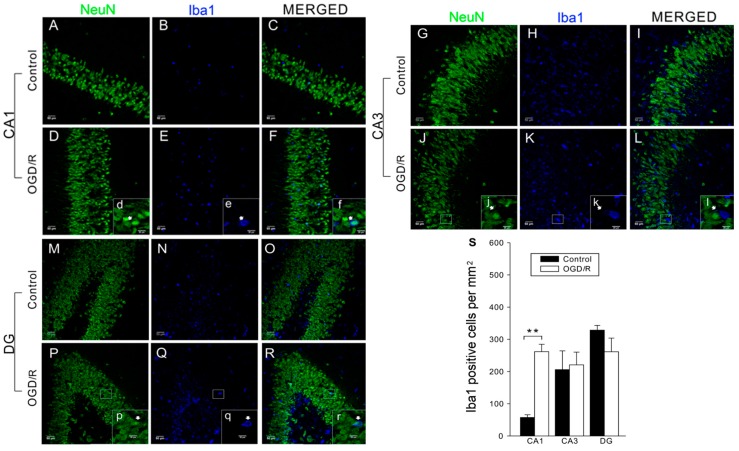
Oxygen–glucose deprivation/reperfusion (OGD/R) promoted microglia activation in hippocampal slices. In each picture, the large area with lowercase letter is enlarged from the small area in white box; the white arrows point at labeled cells and the corresponding area. (**A**–**R**) Examples of neuron-specific nuclear protein (NeuN)-labeled neurons (**green**) and ionized calcium binding adapter molecule 1 (Iba1)-labeled microglia (**blue**). Scale bar: 50 μm; (**D**–**R**) scale bar: 20 μm; (**S**) the density of Iba1-positive cells (cells/mm^2^) in CA1, CA3, and dentate gyrus (DG) before and after OGD/R. The data are expressed as the mean ± standard error of the mean (SEM) (*n* = 5–8). * *p* < 0.05, ** *p* < 0.01.

Result of Western blotting had indicated that OGD/R treatment significantly enhanced expression of Iba1 in cultured primary microglia [17]. To investigate the level of microglial activation in OHSCs, firstly we carried out Iba1 immunofluorescence labeling to identify activated microglia. Iba1-positive cells were found throughout the CA1, CA3, and DG (Figure 4). After OGD/R treatment, the number of Iba1-positive cells increased significantly in the CA1 region compared with the control group (Figure 4A–F,S; *p* < 0.01). However, the number of Iba1-positive cells was not significantly changed upon OGD/R treatment in the other two regions (Figure 4G–S; *p* > 0.05).

**Figure 5:**
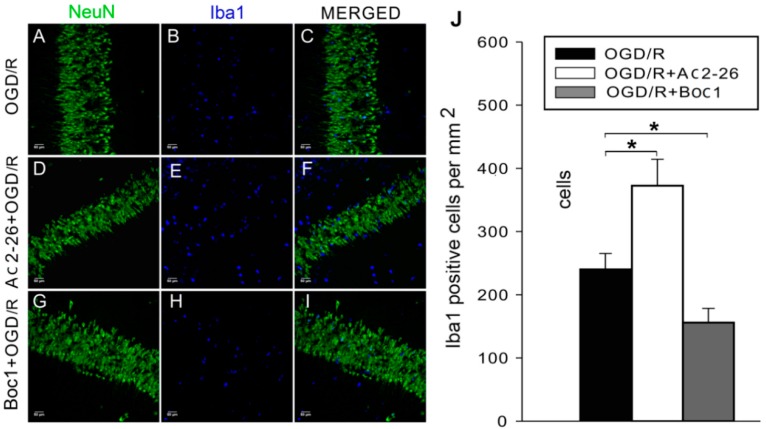
The extent of microglial activation induced by oxygen–glucose deprivation/reperfusion (OGD/R) was altered by Ac2-26 and Boc1 treatment. (**A**–**I**) Examples of neuron-specific nuclear protein (NeuN)-labeled neurons (**green**) and ionized calcium binding adapter molecule 1 (Iba1)-labeled microglia (**blue**). Scale bar: 50 μm; (**J**) after OGD/R treatment, the number of Iba1-positive particles was increased by Ac2-26 treatment (1 μM) and significantly decreased by Boc1 (4 μM) in CA1. The data are expressed as the mean ± standard error of the mean (SEM) (*n* = 6–8). * *p* < 0.05.

We next sought to determine whether (1) exogenous ANXA1 treatment enhances OGD/R-microglial activation; and (2) ANXA1 acts via FPRs. First, we treated OGD/R and control slices with the ANXA1 N-terminal derived peptide Ac2-26, which mimics full length ANXA1. The addition of Ac2-26 during OGD led to an increase in Iba1 expression (Figure 5A–F,J; *p* > 0.05). To assess roles for FPRs in ANXA1-mediated microglial activation, we treated OSHCs with the non-selective FPR antagonist Boc1. Boc1 treatment led to a significant decrease in Iba1 expression compared with the non-treated OGD/R control group (Figure 5A–C,G–J; *p* < 0.01).

**Figure 6:**
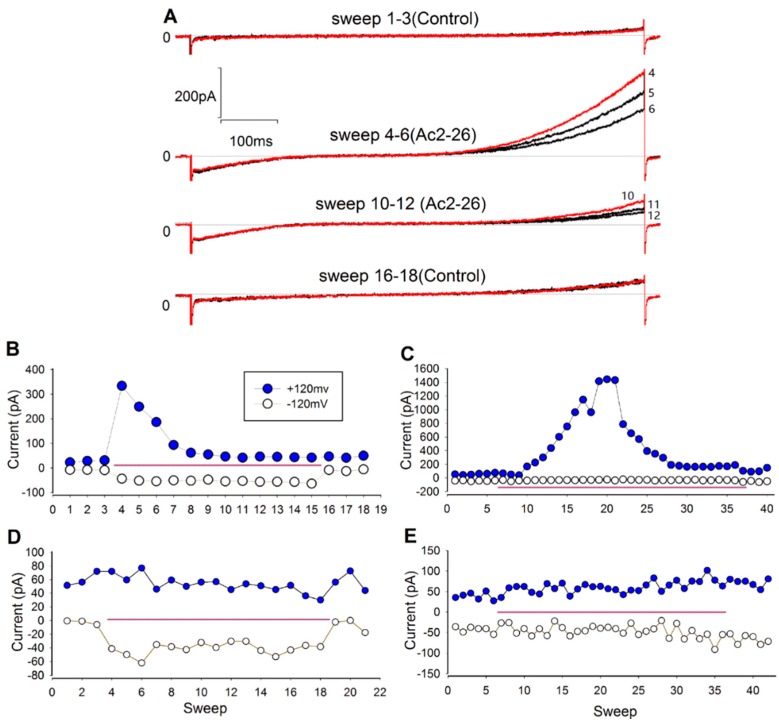
Ac2-26 treatment increased the amplitude of cell membrane currents in BV-2 cells. (**A**) Representative current traces illustrate that Ac2-26 (3.3 μM) increased both the inward current (I*_i_*) and the outward current (I*_o_*). The stimulus protocol was ramp voltage (Figure S2); the red lines are first trace in the groups; (**B**–**E**) the instantaneous values at +120 and −120 mV from the current curves recorded using ramp voltage indicate different types of responses to Ac2-26 treatment (3.3 μM). Pink lines mark the period of Ac2-26 application in the bath; (**B**) both I*_i_* and Io were significantly increased after Ac2-26 treatment; (**C**) example of a cell in which only Io was significantly increased after Ac2-26 treatment; (**D**) example of a cell in which only I*_i_* was significantly increased after Ac2-26 treatment; (**E**) example of a cell in which neither I*_i_* nor Io were significantly affected by Ac2-26 treatment.

Given that activity of microglial ion channels has been reported to play important roles in microglia activation and migration [18], we next carried out whole-cell patch-clamp recordings to determine whether ANXA1 affects the activity of ion channels in the cellular membrane of BV-2 microglia. The BV-2 microglia cell line is regarded as a valid substitute for primary microglia in many experimental settings, including complex cell-cell interaction studies [19]. Step voltage and ramp voltage recordings were obtained to characterize membrane currents. We found that the currents were activated when the membrane potential was higher than approximately +40 mV or lower than approximately −80 mV. The currents of all recorded cells were not significantly affected by Ac2-26 treatment when the voltage levels were in the range of −40 to +20 mV (Figure 6A). At higher and lower voltage ranges, individual cells displayed variable responses to Ac2-26 treatment. In 8 of the cells, both outward currents (I*_o_* activated by positive membrane potential) and inward currents (I*_i_*, activated by negative membrane potential) were significantly elevated after Ac2-26 treatment (Figure 6B); in 9 cells, significant increases were observed only in I_o_ (Figure 6C); in four cells, significant increases were observed only in I*_i_* (Figure 6D); and, in 14 cells, neither I*_o_* nor I*_i_* were significantly affected by Ac2-26 (Figure 6E). Thus, 60% (21/35) of BV-2 microglia displayed an electrophysiological response to Ac2-26.

In summary, immunofluorescence assays indicated that ANXA1 enhances microglial activation via FPR signaling. Electrophysiological differences were observed in response to the Ac2-26 ANXA1 mimetic in BV-2 microglia, which is consistent with our findings and previous reports that cell membrane channel activity mediates microglial activation and migration.

### 2.4. ANXA1 Promotes Migration of BV-2 Microglia via FPRs

**Figure 7:**
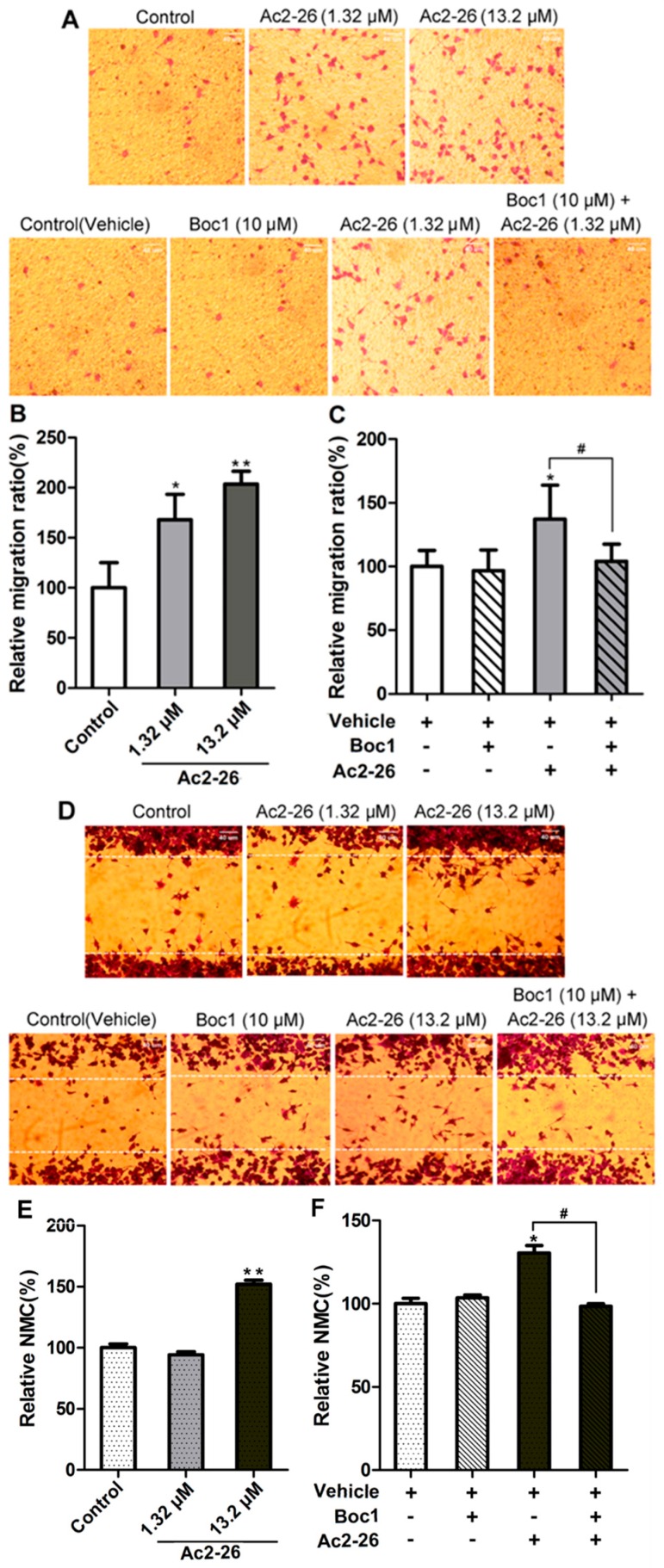
Annexin-1 (ANXA1) mediates BV-2 migration via formyl peptide receptors (FPRs). (**A**) Photographs illustrating the effect of Ac2-26 (1.32 μM) and Boc1 (10 μM) on BV-2 cell migration in trans-well migration assays. Photographs were taken from the lower sides of the chamber’s membrane. Violet particles represent cresyl violet-stained BV-2 cells; (**B**) the relative migration ratio as a percentage relative to the control group; (**C**) the relative migration ratio as a percentage relative to the control + dimethyl sulfoxide (DMSO) group; (**D**) photographs illustrating the effect of Ac2-26 (1.32 μM) and Boc1 (10 μM) on BV-2 cell migration in scratch wound assays. Cells between the two white dotted lines were considered migrating cells; (**E**) the relative number of migrating cells (NMC) as a percentage relative to the control group; (**F**) the relative number of migrating cells (NMC) as a percentage relative to the control + DMSO group. BV-2 cells were cultivated under normal conditions for these experiments. All data are expressed as the mean ± standard error of the mean (SEM) (*n* = 5–15). * *p* < 0.05, ** *p* < 0.01 (compared to the control group), # *p* < 0.05.. Scale bar: 40 μm.

We carried out trans-well migration assays to explore whether ANXA1 mediates microglial migration. BV-2 microglia cells were plated at equal density in the top and bottom wells of a Boyden chamber, and Ac2-26 (1.32 μM) was added to the bottom chamber. BV-2 microglia were cultivated under normal cell culture conditions to promote a resting state. We found that migration of these quiescent BV-2 microglia was significantly increased after Ac2-26 treatment compared with the control groups (Figure 7A–C; *p* < 0.05). To assess potential roles for FPRs in mediating migration, we added Boc1 (10 μM) to the media. Boc1 treatment did not lead to a change in the migration ratio when compared to the vehicle control (Figure 7A,C; *p* > 0.05). However, Boc1 did significantly inhibit Ac2-26-induced migration of BV-2 microglia (Figure 7A,C; *p* < 0.05). We next carried out scratch wound assays to examine the motility of microglia, and found that neither Ac2-26 (1.32 μM) nor Boc1 (10 μM) treatment affected the number of BV-2 microglia infiltrating the scratch wound area under normal conditions (Figure 7D–F; *p* > 0.05). However, a higher concentration of Ac2-26 treatment (13.2 μM) significantly increased the number of migrated BV-2 microglia, and Boc1 treatment significantly inhibited this Ac2-26-induced migration of BV-2 microglia (Figure 7D–F; *p* < 0.05). These findings indicate that ANXA1 promotes migration of BV-2 microglia via FPRs under normal culture conditions.

**Figure 8:**
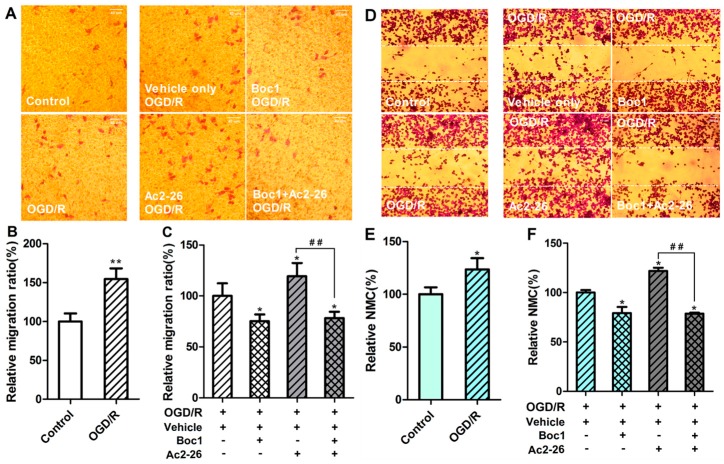
Annexin-1 (ANXA1) promotes BV-2 migration via formyl peptide receptors (FPRs) during oxygen–glucose deprivation/reperfusion (OGD/R). (**A**) Photographs illustrating the effect of OGD/R, OGD/R with Ac2-26 (1.32 μM), or Boc1 (10 μM) on the migration of BV-2 cells in trans-well migration assays. Photographs were taken from the lower sides of the chamber’s membrane, and the violet particles represent BV-2 cells; (**B**) the relative migration ratio as a percentage relative to the control group; (**C**) the relative migration ratio as a percentage relative to the OGD/R + dimethyl sulfoxide (DMSO) group; (**D**) photographs illustrating the effect of OGD/R, OGD/R with Ac2-26 (1.32 µM), or Boc1 (10 μM) on the migration of BV-2 cells in scratch wound assays. The particles between the two white dotted lines were considered migrating cells; (**E**) the relative number of migrating cells (NMC) as a percentage relative to the control group; (**F**) the relative number of migrating cells (NMC) as a percentage relative to the OGD/R + DMSO group; All data are expressed as the mean ± standard error of the mean (SEM) (*n* = 5–10). * *p* < 0.05 (compared to the first group). ** *p* < 0.01 (compared to the control group). ## *p* < 0.01. Scale bar: 40 μm.

Next, trans-well migration and scratch wound assays were used to examine whether ANXA1-mediated FPR signaling promotes microglial migration in response to OGD/R. Trans-well migration assays revealed that the BV-2 migration ratio after OGD/R treatment was significantly increased compared to the control group (Figure 8A,B; *p* < 0.05). Furthermore, Ac2-26 treatment during the OGD period led to an increase in the migration ratio (Figure 8A,C; *p* > 0.05). In contrast, Boc1 treatment (10 μM) over the entire OGD/R period caused a significant decrease in the migration ratio compared to the OGD/R control group (Figure 8A,C; *p* < 0.05). The migration ratios of the Boc1 treatment group and Boc1 + Ac2-26 treatment group were not significantly different (Figure 8A,C; *p* > 0.05). Similarly, scratch wound assays revealed that the number of migrating BV-2 microglia was significantly increased after OGD/R treatment compared to the control group (Figure 8D,E; *p* < 0.05). Ac2-26 treatment during the OGD period led to an increase in the number of migrating cells (Figure 8D,F; *p* < 0.05). As in the trans-well migration assays, Boc1 treatment over the entire OGD/R period led to a significant decrease in the number of migrating cells compared to the OGD/R group (Figure 8D,F; *p* < 0.05). The numbers of migrating cells in the Boc1 treatment group and Boc1 + Ac2-26 treatment group were not significantly different (Figure 8D,F; *p* > 0.05). Taken together, our data indicate that ANXA1 promotes migration of BV-2 microglia in response to OGD/R via an FPR-dependent mechanism.

### 2.5. CK2 Is Involved in ANXA1-Mediated Migration of BV-2 Microglia

**Figure 9:**
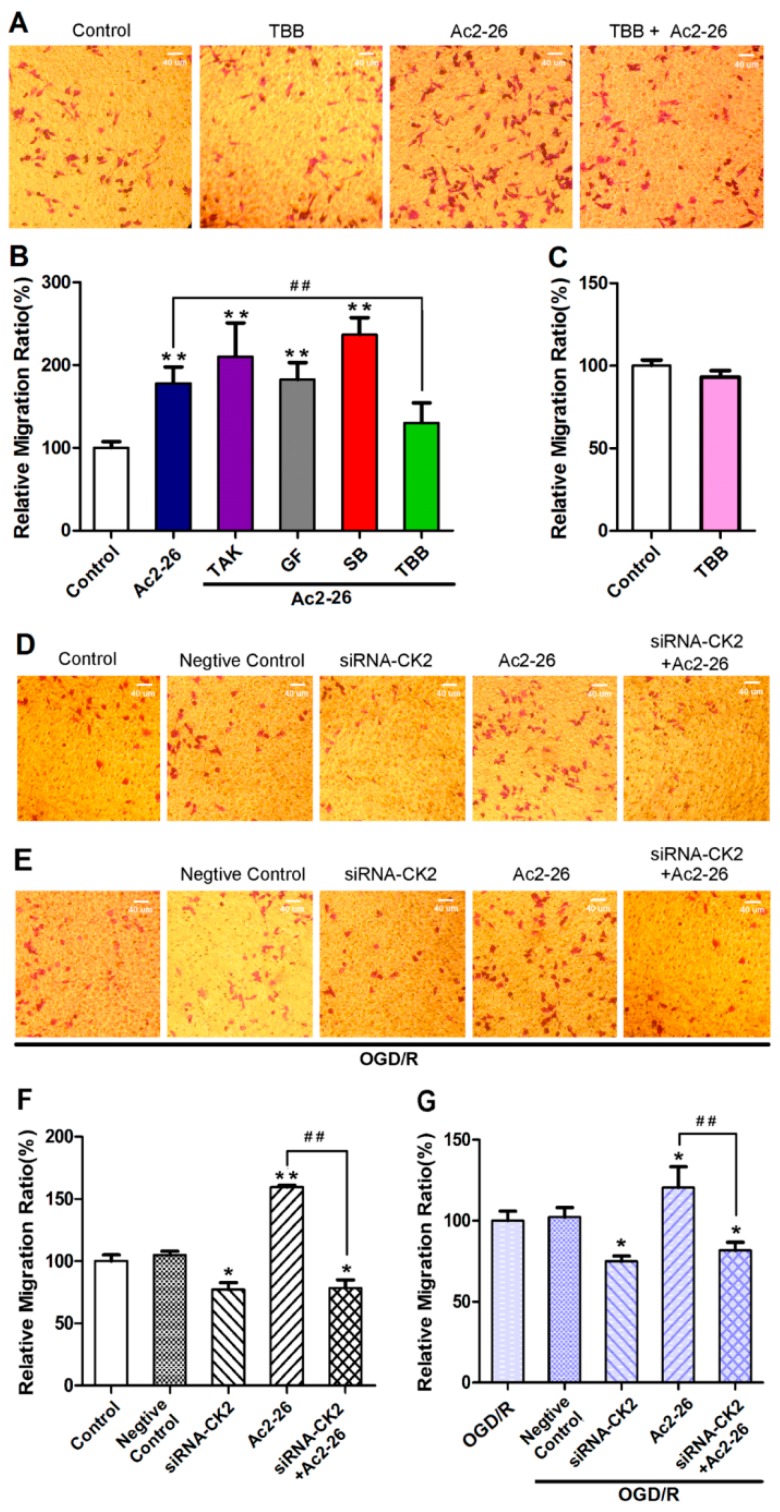
CK2 (casein kinase II) is involved in Ac2-26-mediated BV-2 cell migration. (**A**,**D**,**E**) Photographs illustrating the effect of TBB and the small interfering RNA (siRNA)-CK2 sequence on BV-2 cell migration in trans-well migration assays. Photographs were taken from the lower sides of the chamber’s membrane; the violet particles represent cresyl violet-stained BV-2 cells; (**B**,**C**,**F**) the relative migration ratio in different groups as a percentage relative to the control group; (**G**) the relative migration ratio in different groups as a percentage relative to the oxygen–glucose deprivation/reperfusion (OGD/R) group. The siRNA sequence was used to inhibit CK2 expression. The final concentration of drugs were as follows: Ac2-26, 1.32 μM; TBB, 40 μM; GF, 2 μM; TAK, 1 μM; SB, 0.1 μM. All data are expressed as the mean ± standard error of the mean (SEM) (*n* = 5–9). * *p* < 0.05, ** *p* < 0.01 (compared to the first group), ## *p* < 0.01. Scale bar: 40 μm.

Trans-well migration assays were further employed to determine which pathway(s) were involved in Ac2-26-mediated migration of BV-2 microglia. TBB, TAK632, GF109203X, and SB202190 were used to inhibit CK2 (casein kinase II), PKC (Protein Kinase C), Raf, and P38, respectively. Among these compounds, only TBB significantly inhibited Ac2-26-mediated migration of BV-2 microglia when cells were cultivated under normal culture conditions (Figure 9A–C). Inhibition of CK2 expression with siRNA led to a significant decrease in spontaneous cell migration and Ac2-26-induced (1.32 μM) cell migration (*p* < 0.05) (Figure 9D,F). When BV-2 microglia were subjected to OGD/R, inhibition of CK2 expression with siRNA similarly led to a significant decrease in cell migration (*p* < 0.05) (Figure 9E,G) in both Ac2-26-treated (1.32 μM) and non-treated cells. Together, these results indicate that CK2 is involved in ANXA1-mediated migration of BV-2 microglia.

### 2.6. CK2 Is Involved in ANXA1-Induced α-E-Catenin Phosphorylation in BV-2 Microglia

**Figure 10:**
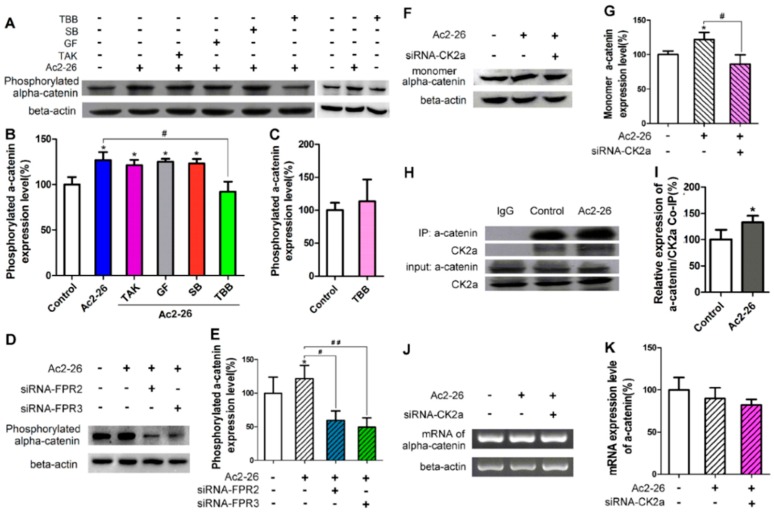
CK2 is involved in Ac2-26-mediated α-catenin phosphorylation in BV-2 cells. (**A**) Western blots illustrating the effects of Ac2-26 and/or the specified inhibitors on phosphorylated α-catenin expression; (**B**,**C**) results of Western blots illustrate the relative expression level of phosphorylated α-catenin as a percentage relative to the control group; (**D**–**G**) results of Western blots illustrate that small interfering RNA (siRNA) inhibition of FPR2, FPR3, and CK2 expression led to down-regulation of Ac2-26-induced (1.32 μM) α-catenin phosphorylation; (**H**,**I**) co-immunoprecipitation illustrates that Ac2-26 treatment (2.64 μM) significantly enhanced the CK2-α-catenin binding; (**J**,**K**) reverse transcription polymerase chain reaction (RT-PCR) results indicate that Ac2-26 treatment or siRNA sequence inhibition of CK2α expression does not affect the expression of total α-catenin. The final concentration of drugs were as follows: Ac2-26, 1.32 μM; TBB, 40 μM; GF, 2 μM; TAK, 1 μM; SB, 0.1 μM. All data are expressed as the mean ± standard error of the mean (SEM) (*n* = 4–12). * *p* < 0.05, (compared to the control groups), # *p* < 0.05, ## *p* < 0.01.

Turnover of substrate adhesion molecules plays critical roles in cell migration [20]. The actin-binding protein α-E-catenin (α-catenin) is an important protein in substrate adhesion structures and CK2 was previously reported to play roles in α-catenin phosphorylation, which leads to the disassociation of α-catenin from β-catenin [21]. We hypothesized that this process also occurs in ANXA1-mediated microglial migration, and found using Western blot analysis that phosphorylated α-catenin expression levels in BV-2 microglia cultivated under normal conditions were significantly enhanced by Ac2-26 treatment (1.32 μM) (Figure 10A,B; *p* < 0.05). Treatment with siRNA sequences to inhibit FPR2 and FPR3 expression led to down-regulation of Ac2-26-induced α-catenin phosphorylation (Figure 10D,E; *p* < 0.05). Expression of FPR1 was not observed in BV-2 microglia. TBB, TAK632, GF109203X, and SB202190 were used to inhibit CK2, PKC, Raf, and P38, respectively. Of these inhibitors, only TBB significantly inhibited Ac2-26-mediated α-catenin phosphorylation levels in BV-2 microglia (Figure 10A–C; *p* < 0.05), which was in keeping with the results of trans-well migration assays shown in Figure 9A–C. Ac2-26 treatment enhanced the expression of the α-catenin monomer (as determined using a monomer specific antibody in western blot analysis), while siRNA inhibition of CK2 expression reversed this effect (Figure 10F,G; *p* < 0.05). Using co-immunoprecipitation, we found that Ac2-26 treatment (2.64 μM) significantly enhanced the interaction of CK2 and α-catenin (Figure 10H,I; *p* < 0.05). RT-PCR analysis demonstrated that Ac2-26 treatment and siRNA inhibition of CK2α expression did not affect the overall expression of α-catenin (Figure 10J,K; *p* > 0.05). Taken together, these results indicate that CK2 is involved in ANXA1-induced α-catenin phosphorylation and disassociation in BV-2 microglia, but not overall α-catenin expression.

## 3. Discussion

ANXA1 had been found expressed in cultured neuron and microglia (including BV-2 microglia), and OGD/R treatment significantly increased the expression in these cells [17,22,23]. In present study we found that, in rat hippocampus ANXA1 was largely expressed in neurons and only rarely ANXA1 particles were observed in microglia, in contrast FPRs were richly expressed in microglia and only rarely in neurons. Both ANXA1 and FPRs might partly derive from astrocytes or oligodendrocytes, it should be clarified by future study. Both neuronal expression of ANXA1 and microglial expression of FPR were increased by OGD/R treatment. Given that ANXA1 is an agonist of FPRs, these findings indicate that ANXA1 binding of FPRs may mediate important crosstalk between neurons and microglia in response to OGD/R. In support of this hypothesis, blocking FPRs with Boc1 during OGD treatment inhibited OGD/R-induced microglial activation in CA1. We also found that the neural injury in CA1 region was exacerbated after OGD/R by doing TUNEL (TdT-mediated dUTP-biotin Nick End Labeling) (Figure S1) assay. FPRs activation (Ac2-26 treatment) significantly decreased the neural injury induced by OGD/R (Figure S1). These findings indicate that ANXA1-FPRs play neural protective roles during OGD/R. Consistent with previous reports that hippocampal CA1 is more sensitive to ischemia than the DG or CA3 [24,25], we found that changes in microglial activation and ANXA1 expression were not significant in CA3 or DG after OGD/R treatment. We have previously shown that OGD/R treatment stimulates ANXA1 expression in primary microglia and upregulates FPR expression; however, ANXA1 was not found to be expressed by cortical neurons derived from fetal Sprague-Dawley rats [17]. In contrast, findings from the present study indicate that ANXA1 was mainly synthesized by neurons in OHSCs from 4-week old rats, suggesting age- and region-specific differences in ANXA1 expression.

In both a previous study by Luo et al. [17] and the present study, we found compelling evidence that ANXA1 mediates microglia and migration of BV-2 microglia via FPRs. OGD/R treatment enhanced Iba1 expression and ANXA1 expression in cultured rat primary microglia [17] and in rat hippocampus slices. We’ve now discovered that the antagonist of FPRs (Boc1) blocked the effect of OGD/R on Iba1 expression. Ac2-26 was found to promote primary microglial migration in a trans-well assay under standard conditions, and this effect was blocked by Boc1 [17]. In this study, Boc1 treatment during OGD/R inhibited migration, and Ac2-26 treatment during OGD exerted the opposite effect. We postulate that ANXA1 and FPRs generally elevate the mobility of microglia and of BV-2 microglia during OGD/R.

Several lines of evidence have demonstrated a neuroprotective effect of ANXA1 in ischemia as well as microglial migration to sites of injury where they can release chemokines and carry out phagocytosis [17,26]. ANXA1 has been shown to regulate the migration of many kinds of cells, such as neutrophils [27] and endothelial cells [28]. ANXA1 was also found to play critical roles in the engulfment and phagocytosis of apoptotic cells [15,16]. It has been well demonstrated that microglia move along chemokine gradients in injury models of autoimmune encephalitis, Alzheimer’s disease, and cerebral ischemia [29]. Based on our results and previous studies, we speculate that ANXA1 is secreted in the OGD/R injury area and may act as a signal that guides microglia to the lesion site. ANXA1 may also guide local migration within the infarct by directing movement toward apoptotic cells, while also carrying out additional anti-inflammatory and repair functions. Further experimental evidence is needed to confirm this speculation.

Changes in membrane potential and ionic currents have been shown to be important for microglial activation and migration [16,30,31,32]. Ac2-26 treatment was found to induce an increase of Ca^2+^ influx in primary microglia, which could be blocked by Boc1 [17]. Accordingly, we found that Ac2-26 treatment increased the amplitude of whole-cell membrane currents in BV-2 microglia. These findings indicated that ANXA1 could trigger rapid membrane and intracellular responses. ANXA1 acts upstream of receptor and intracellular signaling processes; therefore, it is possible that these processes represent initial responses that direct microglial phenotype conversion.

BV-2 microglia proliferate rapidly and are very sensitive to environmental change. Therefore, microglia constitute a dynamic population that may exhibit markedly different properties depending on their particular state. In our whole-cell patch-clamp recordings, we observed that electrophysiological properties varied markedly from cell to cell. For instance, different BV-2 microglia displayed significantly different current amplitudes and electrophysiological responses to Ac2-26 treatment (Figure 6B–F). This finding may be due to differences in channel properties or differential expression of regulatory proteins on the cell membrane. Our finding that Ac2-26 alters the amplitude of whole-cell membrane currents in BV-2 microglia indicates that ANXA1 plays indirect roles in regulating the activity of ion channels on the microglial cell membrane, which may be important for microglial activation and migration. The specific channels underlying ANXA1-induced electrophysiological responses will be the topic of future studies.

Our data indicate that ANXA1 acts upstream of FPRs and CK2 signaling. ANXA1 is a known agonist of FPRs [14], of which FPR1, FPR2, and FPR3 are the major members. Consistent with a downstream role of ANXA1 in neuronal injury responses, both Ac2-26 and conditioned medium isolated from neurons after exposure to OGD/R up-regulated the expression of FPR1, FPR2, and FPR3 in microglia [17]. Previous studies have demonstrated an interaction between FPRs and cytoskeletal proteins [33,34], suggesting that cytoskeletal rearrangement is critical for microglia migration [35]. This finding suggests that ANXA1 may mediate microglial migration by regulating cytoskeletal dynamics through FPRs.

This result raises the intriguing question of which cytoskeletal proteins may be regulated downstream of ANXA1. Many proteins, including F-actin, actin-regulatory molecules (Arp2/3), talin, and vinculin, are known to participate in the construction of the leading edge of migrating cells (lamellipodia or filopodia) [35,36]. Orai1, STIM1, and several Ca^2+^ responsive proteins (SK3, CaM, and Iba1) have also been found to be important components of the lamellipodia of microglia [35]. In addition to the generation of a leading edge, cell migration and invasion through tissue requires turnover of adherent substrates that enable forward-propelling machinery disassembly in the rear and reassembly in newly protruding sites [20]. Among these, α-catenin is an actin-binding protein in adhesive structures that connects microfilaments and associated proteins (e.g., vinculin and α-actinin) to β-catenin. In turn, β-catenin is connected to cadherins, which are trans-membranous cell adhesion molecules [37,38,39]. In addition, as part of the Wnt pathway, free β-catenin is taken into the nucleus where it binds LEF1, a transcriptional activator [40]. Mutations in α-catenin genes lead to defects in cell adhesion molecules and Wnt pathway signaling [41]. We found that Ac2-26 treatment enhanced the expression of phosphorylated α-catenin, thereby providing preliminary evidence for a connection between ANXA1-mediated microglial migration and α-catenin-dependent cytoskeletal dynamics.

How OGD/R and ANXA1 regulate α-catenin is interesting and is to be explored by further research. In this study, we found that ANXA1 may regulate α-catenin via the activation of the protein kinase CK2, which is a ubiquitous eukaryotic messenger-independent serine/threonine kinase. Studies have demonstrated that CK2 binds with members of the Wnt pathway, many of which are substrates of CK2, including β-catenin [21,42,43,44]. In the present study, inhibition of CK2 with TBB or siRNA sequence blocked α-catenin phosphorylation in BV-2 cells and inhibited Ac2-26-mediated migration of BV-2 microglia. These findings indicate that the CK2 pathway plays important roles in regulating microglial migration. Consistent with this possibility, α-catenin phosphorylation mediated by CK2 phosphorylation (on the α-subunit, primarily at T360/S362) promotes β-catenin transactivation and tumor cell invasion [21]. It remains to be clarified whether a similar mechanism exists in ANXA1-mediated microglia migration. Future study should focus more attention on activity upstream and downstream of CK2 and α-catenin under OGD/R-induced and ANXA1-mediated cell migration.

## 4. Material and Methods

All experimental protocols were approved by the Tongji Medical College, Huazhong University of Science and Technology, and the methods were carried out in accordance with the approved guidelines and regulations.

### 4.1. Animals

All animal experiments were performed in accordance with Guide for the Care and Use of Laboratory Animals (Institute of Laboratory Animal Resources, Commission on Life Sciences, National Research Council; NIH Pub. No. 85-23, revised 1996). They were approved by Animal Care and Use Committee of Huazhong University of Science and Technology (SYXK:2010-0057, 25 October 2010), and all efforts were made to minimize suffering. Rat organotypic hippocampal slice cultures (OHSCs) were derived from four-week-old male (*n* = 25) and female (*n* = 25) Sprague-Dawley rats. The animals were supplied by the Experimental Animal Center of Tongji Medical College of Huazhong University of Science and Technology (SCXK:2010-0009), Wuhan, China, and were housed in individual cages exposed to a 12-h light/dark cycle with food and water available ad libitum.

### 4.2. Reagents

The ANXA1 N-terminal peptide Ac2-26 (acetyl-AMVSEFLKQACYIEKQEQEYVQAVK) was synthesized by the AngTai Biotechnology Center (Hangzhou, China) based on previously published data. The non-selective FPR antagonist Boc1 (N-t-Boc-Phe-Leu-Phe-Leu-Phe) was purchased from Calbiochem (San Diego, CA, USA). The CK2 inhibitor TBB (4,5,6,7-tetrabromobenzotriazole) was supplied by Merck (Whitehouse Station, NJ, USA). GF109203X (GF), TAK632 (TAK), and SB202190 (SB) (inhibitors of the PKC pathway, Raf pathway, and P38 pathway, respectively) were supplied by Selleck Chemicals (Houston, TX, USA). Boc1 and stock solutions of the chemical inhibitors were prepared by dissolving the compounds in dimethyl sulfoxide (DMSO). The final concentrations of Ac2-26 and Boc1 were determined by preliminary experiments, and the final concentrations of the inhibitor stocks were determined according to the manufacturer’s protocol. In experiments where BV-2 microglia were treated with both Ac2-26 and an inhibitor, cells were first treated with the inhibitor for more than 2 h before treatment with Ac2-26. In all experiments, the corresponding no-drug control group received the same volume of vehicle as the experimental group. Small interfering (si) RNA duplex sequences and the negative control sequence were synthesized by Shanghai GenePharma Co., Ltd., (Shanghai, China).

### 4.3. Rat Organotypic Hippocampal Slice Cultures

Rat cultured OHSCs were prepared as previously described [45,46]. Briefly, 350-μm-thick transverse hippocampal slices were prepared from 28- to 30-day-old Sprague-Dawley rats using a McIlwain tissue chopper (Campden Instruments, Leicester, UK) in pathogen-free aseptic environment (when chopping the bains and the slices were soaked in ice-cold D-Hanks balanced salt solution supplemented with 1% (*v*/*v*) penicillin/streptomycin). Slicees were then transferred to a humidified semi-porous membrane (30 mm Millicell-CM tissue culture plate insertswith 0.4-μm pore) (Millipore, Rome, Italy), which was placed in 6-well tissue culture plates (6 slices per membrane). Each well contained 1.2 mL of tissue culture medium, and the medium was composed of 75% glucose-containing Dulbecco’s modified Eagle’s medium (DMEM) and 25% heat-inactivated horse serum. OHSCs were maintained at 37 °C and 100% humidity in a 5% CO_2_ and air atmosphere.

As for OGD/R treatment to OHSCs, firstly the OHSCs were transiently maintained in 6-well plates containing 1.2 mL of glucose-free DMEM under anoxic conditions (5% CO_2_, 94% N_2_, 1% O_2_, 37 °C) for 2 h (OGD 2 h). After OGD, slices were returned to normal conditions (e.g., standard glucose and O_2_ levels) for an additional 24 h. In the control OHSCs, standard culture medium was replaced for 2 h and then again replaced with standard medium for an additional 24 h. Outside of the OGD period, slices were kept in normal tissue culture conditions: 37 °C, 5% CO_2_, and 95% atmospheric air.

### 4.4. OGD/R Treatment of BV-2 Microglia

After the cells were plated, BV-2 cultures were transferred to an anaerobic incubator with 5% CO_2_ and 95% atmospheric air, and cultivated at 37 °C (i.e., normal conditions) for 24 h. Then, the cells were washed carefully three times with phosphate-buffered saline (PBS; 0.1 M; pH 7.4), the medium was changed to glucose-free DMEM, and the cultures were subjected to anoxic conditions for 2 h (OGD). Following OGD treatment, the medium was replaced with high-glucose DMEM (standard medium) and the cultures were returned to normal conditions for 24 h. After this period, cells were collected for trans-well migration assays, scratch wound assays, or western blot analysis.

### 4.5. Primary Antibody and Immunofluorescence

Slices were fixed in 4% paraformaldehyde in phosphate buffer (PB; 0.1 M; pH 7.4 without NaCl) for 6 h at 4 °C and, after three washes in PB, slices were transferred to 30% sucrose in PB solution at 4 °C and left until the specimens were submerged. Sections of 30-μm thickness were obtained using a freezing microtome on poly-l-lysine-coated slides. The sections were then rinsed three times in PBS (0.1 M; pH 7.4) for 5 min each. After 30 min of pre-incubation in blocking buffer (10% normal donkey serum and 0.2% Triton X-100 in PBS), sections were incubated for 24 h with primary antibodies diluted in PBS. The following primary antibodies were used: mouse monoclonal antibody [1B7] targeted to neuron-specific nuclear protein (NeuN) to label neurons (Anti-NeuN; 1:50 dilution; Millipore, Billerica, MA, USA); goat polyclonal antibody targeted to ionized calcium binding adapter molecule 1 (Iba1) (Anti-Iba1; 1:100 dilution; Abcam, Cambridge, UK) or rabbit polyclonal antibody targeted to Iba1 (Anti-Iba 1; 1:250 dilution; Wako, Osaka, Japan) and mouse monoclonal antibody targeted to OX-42 (a monoclonal antibody to CD11b) (Anti-OX-42; 1:100 dilution; Abcam) to label activated microglia; and rabbit polyclonal anti-annexin-1 (anti-annexin-1 (H-65); 1:50 dilution; Santa Cruz Biotechnology, Inc., Santa Cruz, CA, USA) and goat polyclonal anti-FPR (anti-FPR (M-20); 1:100 dilution; Santa Cruz Biotechnology, Inc.).

To perform triple immunofluorescence assays, we incubated sections with antibodies for NeuN and Iba1 together with FPR or ANXA1. Thereafter, sections were incubated for 1 h at room temperature in a solution containing an appropriate mixture of the following secondary antibodies: Texas Red-conjugated donkey anti-rabbit IgG (1:500 dilution; Abcam), DyLight^®^ 405-conjugated donkey anti-goat IgG (1:100 dilution; Jackson ImmunoResearch, West Grove, PA, USA), and DyLight^®^ 488-conjugated donkey anti-mouse IgG (1:500 dilution; Abcam). Finally, any excess liquid from the specimens was removed by tapping the side of the slide onto a clean laboratory wipe, after which one drop of anti-fade reagent (Invitrogen, Carlsbad, CA, USA) was applied to the specimen. Next, specimens were slide-mounted with a coverslip, and immunofluorescence imaging was performed using the NIH Image J version 1.47 software program (National Institutes of Health, Bethesda, MD, USA).

### 4.6. Whole-Cell Patch Clamp Recordings

Whole-cell patch-clamp recordings were conducted on the soma of BV-2 microglia. During the experiments, the temperature was maintained at 25 °C. Before the electrophysiology experiments, BV-2 microglia were plated on coverslips placed in 24-well plates filled with 500 μL of DMEM containing 10% fetal bovine serum (FBS) (*v*/*v*), and incubated an anaerobic incubator with 5% CO_2_ and 95% atmospheric air, and cultivated at 37 °C (i.e., normal conditions) for 24 h. Culture dishes of 35 mm diameter were filled with 1.5 mL artificial cerebrospinal fluid (ACSF) and placed on the stage of an inverted microscope Olympus IX71 (Olympus, Tokyo, Japan). The ACSF was composed of 130 mM NaCl, 5 mM KCl, 1 mM MgCl_2_, 1 mM CaCl_2_, 10 mM HEPES, 10 mM EGTA, and 5 mM glucose (pH 7.2, 280–300 mOsm) [42]. The coverslips containing cells were placed in the center of the dishes. Recording electrodes (3–6 MΩ) contained a K^+^-based internal solution composed of (in mM): 100 K-gluconate, 40 KCl, 1 MgCl_2_, 1 CaCl_2_, 10 HEPES, and 2 ATP-Na_2_ (pH 7.2, 280–300 mOsm) [47]. Electrodes were pulled with a PUL-2 pipette puller (World Precision Instruments Inc., Sarasota, FL, USA) and were precisely controlled with a Burleigh PCS-5000 micromanipulator (Thor Labs, Newton, NJ, USA) during the experiments. Data were amplified and filtered at 2 kHz by an Axon patch 700B patch-clamp amplifier (Molecular Devices, Sunnyvale, CA, USA), digitized by a Digidata 1440A (Molecular Devices), stored, and analyzed by pCLAMP (Molecular Devices). We used ramp as a stimulus voltage protocol (Figure S2). The scanning voltage was from −140 to +120 mV and the scanning time was 1 s. Between two scanning periods, the membrane potential was held at −20 mV [48].

For Ac2-26 treatment during electrophysiology, Ac2-26 was diluted in ACSF to a final concentration of 3.3 μM. The solution was filled into an injector, which was connected to the culture dishes by thin plastic pipes. The inner diameter of the pipes was approximately 0.3 mm, and the diameters of the tips were approximately 100 μM (10 pipe tips were placed side by side, and then pulled at high temperature). Gravity forced the fluid in the injectors to flow out of the tips, thus creating a bath solution of Ac2-26. As a control, ACSF containing no Ac2-26 was applied in the same manner.

### 4.7. Trans-Well Migration Assays

Before BV-2 microglia were plated, Boyden chambers (8-μm pore size, 24-well inserts, Corning Incorporated, Corning, NY, USA) were placed in DMEM containing 0.1% bovine serum albumin (BSA) and incubated at 37 °C, 5% CO_2_ for 2 h. An equal number of cells (1 × 10^4^ cells) were plated on the top chamber with DMEM containing 0.1% BSA. DMEM with 10% FBS was placed in the lower well. For experiments using Ac2-26 and Boc1, media in the lower chambers were supplemented at the following concentrations: 1.32 μM for Ac2-26 and 10 μM for Boc1. After 24 h of incubation, media in the upper and lower wells were discarded, and the cells were fixed with 4% paraformaldehyde for 20 min and then stained with 0.1% crystal violet for 15 min. After being carefully washed with PBS three times, cells attached to the membranes were photographed at 100× magnification using an inverted microscope Olympus IX71 (Olympus, Tokyo, Japan) equipped with a camera Samsung S860 (8,100,000 pixels; Samsung, Seoul, Korea). Cells that did not migrate through the pores on the upper surface of the insert membranes were removed using a cotton swab, and the membranes were photographed again to record the migrating cells. For each membrane, five random fields of view were photographed (i.e., 15 fields of view per well were recorded). The cells (i.e., crystal violet particles) in the photographs were counted. The number of cells that attached on the lower surface of the membrane was defined as N_1_ and the number of cells attached to the membrane of the inserts was defined as N_2_. Due to difficulty in distinguishing cells on the upper versus lower surface of the membranes, the proportion of migrating cells, i.e., the migration ratio (MR), was also calculated as N_1_/N_2_ × 100%.

### 4.8. Scratch Wound Assays

BV-2 microglia were seeded at 2 × 10^4^ cells per well in 24-well plates, and cultured with DMEM containing 10% FBS under normal conditions until cells were approximately 95% confluent (approximately 1 day). The resulting monolayer of cells was washed with PBS and then scratched with a sterile 200-μL pipette tip. Next, the medium was replaced with DMEM containing no FBS (high glucose for control treatments or glucose-free for OGD/R). Ac2-26 (1.32 μM), Boc1 (10 μM), or DMSO (i.e., the vehicle control) was then added to the media. The cells were incubated for 24 h, then fixed with 4% paraformaldehyde for 20 min and stained with 0.1% crystal violet for 15 min. After washing three times with PBS, three random fields along the region of the scratch were photographed as described for the trans-well migration assays. The crystal violet-stained cells that infiltrated the wound areas were counted as the number of migrating cells (NMC).

### 4.9. Transfection of BV-2 Microglia with Small Interfering (si) RNA

BV-2 microglia were plated in 6-well plates (Corning Inc., Corning, NY, USA) in DMEM (Invitrogen) containing 10% FBS (Gibco, Invitrogen, Carlsbad, CA, USA) at 3 × 10^5^ cells/well for 24 h before transfection in a humidified atmosphere of 5% CO_2_ and 95% air at 37 °C (in trans-well migration assays, the cells were plated in Boyden chambers at 1 × 10^4^ cells/well as described above). Then, the medium was changed to DMEM containing no FBS, and the cells were transfected with siRNA sequences or the control sequence for 6 h following the suggestion of the individual who provided the sequences (GenePharma Co., Ltd., Shanghai, China). The transfected cells were harvested after 24 h of incubation. For nFPR siRNA transfection, pre-validated siRNA duplex sequences selected for use [14] from the three that underwent initial evaluation are shown in Table 1. For CK2 siRNA transfection, the siRNA duplex sequence (Csnk2a2-Mus-543, shown in Table 1) was pre-validated by western blot to insure that CK2 expression could be decreased by it at least 50%.

### 4.10. Western Blotting

Our protocol for western blotting was the same as previously described [17]. Briefly, after gel transfer, membranes were washed with Tris-buffered saline containing 0.1% Tween (TBST) and incubated with 1:1000 anti-p-α-E-catenin (Ser-641) (Santa Cruz Biotechnology, Inc.) in TBST containing 5% BSA overnight at 4 °C. After three 10-min washes with TBST, the membranes were incubated with secondary antibody (horseradish peroxidase-conjugated, 1:20000 dilution in TBST) (Santa Cruz Biotechnology, Inc.) and then exposed to film after chemiluminescence treatment. All procedures were carried out at room temperature (approximately 25 °C) unless otherwise noted. Scanned images of the developed blots were quantified using Kodak Image Analysis software (Kodak Digital Science 1D, Eastman Kodak Company, Rochester, NY, USA).

### 4.11. Co-Immunoprecipitation

The interaction between CK2α and α-catenin was confirmed by co-immunoprecipitation assay. BV-2 microglia were harvested after 24 h of Ac2-26 treatment (6.6 μM). After three washes with pre-chilled PBS, cold radio-immunoprecipitation assay (RIPA) lysis buffer was added to the plate (1 mL for a 6-cm plate) for 15 min. Cells were scraped into clean 1.5-mL Eppendorf tubes with a clean cold scraper, and then centrifuged at 12,000 rpm at 4 °C for 15 min. The supernatant was immediately transferred to new tubes. The total protein was quantified with BCA assay. For immunoprecipitation, 800 μg of the cell lysate was incubated with CK2 antibody (3 μL) or Iba1 antibody (3 μL) overnight at 4 °C on a rotator. Protein A/G-agarose was added and incubated at 4 °C on a rotator for 3 h. After centrifugation at 2500 rpm at 4 °C for 5 min, the supernatant was absorbed, and protein A/G-agarose beads were washed three times with PBS. The antigen-antibody complex were incubatedat 95 °C for 5 min after 2× loading buffer (same volume as protein A/G-agarose) was added to the sediment. Immunoprecipitated proteins were resolved by polyacrylamide gel electrophoresis (SDS-PAGE) followed by western blot analysis. Antibodies used for immunoprecipitation included: anti-CK2α, anti-α-catenin, and anti-Iba1 (Santa Cruz Biotechnology, Inc.). Relative intensities were calculated after normalization with total protein using the software ImageJ1.48u (National Institutes of Health, Bethesda, MD, USA).

### 4.12. RT-PCR

Reverse transcription polymerase chain reaction (RT-PCR) analysis with gene-specific primers was used to detect α-catenin mRNA in BV-2 microglia. Cells were collected by centrifugation at 12,000 rpm for 5 min and then prepared with an RNeasy mini total RNA preparation kit (Qiagen, Shanghai, China). Reverse transcription (RT) was performed with 1 μg of total RNA using M-MLV reverse transcriptase. One µL of the RT product was added to the reaction mixture containing 1× PCR buffer (10 mM Tris-HCl, pH 8.3, 50 mM KCl), 1.5 mM MgCl_2_, 0.2 mM dNTPs, and 2.5 units of Taq polymerase for 30 min at 42 °C. The PCR primer sequences used were as follows: β-actin rat) forward: 5′-CACCCGCGAGTACAACCTTC-3′ and reverse: 5′-CCCATACCCACCATCACACC-3′; and α-catenin (mouse) forward: 5′-GAGATGACCGACTTCACCCG-3′ and reverse: 5′-GACGACCAGCTCTCCACCAA-3′. Agarose gel electrophoresis was used to detect the products of PCR amplification, the sample volume for each PCR product was 10 μL, the concentration of agarose gel was 2%, and ethidium bromide was used for staining.

### 4.13. Statistical Analysis

Immunofluorescence images were analyzed using the NIH ImageJ version 1.47 software program (National Institutes of Health). Data were expressed as the mean ± standard error of the mean (SEM). The data were analyzed with SPSS v16 software (SPSS Inc., Chicago, IL, USA). One-way ANOVA and Student’s *t*-test were used to determine statistically significant differences between the values of various experimental groups. A *p*-value <0.05 was considered statistically significant. Statistical figures were generated using GraphPad Prism 5 (GraphPad Software Inc., La Jolla, CA, USA).

## 5. Conclusions

In conclusion, we found that expression of ANXA1 and FPRs were increased after OGD/R treatment in hippocampal slices. ANXA1 promoted microglial activation and migration of BV-2 microglia via FPRs during OGD/R. Both α-catenin phosphorylation and the CK2 pathway were implicated in the ANXA1-mediated response. The specific mechanisms by which ANXA1 affects these molecular processes and their precise roles in OGD/R will be clarified in future work.

## Figures and Tables

**Table 1:** Sequences for small interfering RNA (siRNA) transfection.

Target Protein	Sense Sequence	Antisense Sequence
FPR1	5′-AGCGAAUACAGCAGGUGUCTT-3′	5′-GACACCUGCUGUAUUCGCUTT-3′
FPR2	5′-GGCUCAAACUGAUGAAGAATT-3′	5′-UCUUCAUCAGUUUGAGCCTT-3′
FPR3	5′-GAGGGAUCAUCAGGUUCAUTT-3′	5′-AUGAACCUGAUGAUCCCUCTT-3′
CK2α	5′-CCAUUAACAUCACCAACAATT-3’	5′-UUGUUGGUGAUGUUAAUGGTT-3′
Negative Control (NC)	5′-UUCUCCGAACGUGUCACGUTT-3′	5′-ACGUGACACGUUCGGAGAATT-3′

## Supplementary Materials

Supplementary materials can be found at www.mdpi.com/1422-0067/17/10/1770/s1.
