# Supplementary material for: Annexin-1 Mediates Microglial Activation and Migration via the CK2 Pathway during Oxygen–Glucose Deprivation/Reperfusion
Source: Int J Mol Sci. 2016 Oct 22;17(10):1770. doi: 10.3390/ijms17101770 (PMC5085794; doi:10.3390/ijms17101770)
Supplement: Supplementary file 1 [file ijms-17-01770-s001.pdf]

Supplementary Materials: Annexin-1 Mediates Microglial Activation and Migration via the CK2 Pathway during Oxygen–Glucose Deprivation/Reperfusion

Shuangxi Liu, Yan Gao, Xiaoli Yu, Baoming Zhao, Lu Liu, Yin Zhao, Zhenzhao Luo and Jing Shi

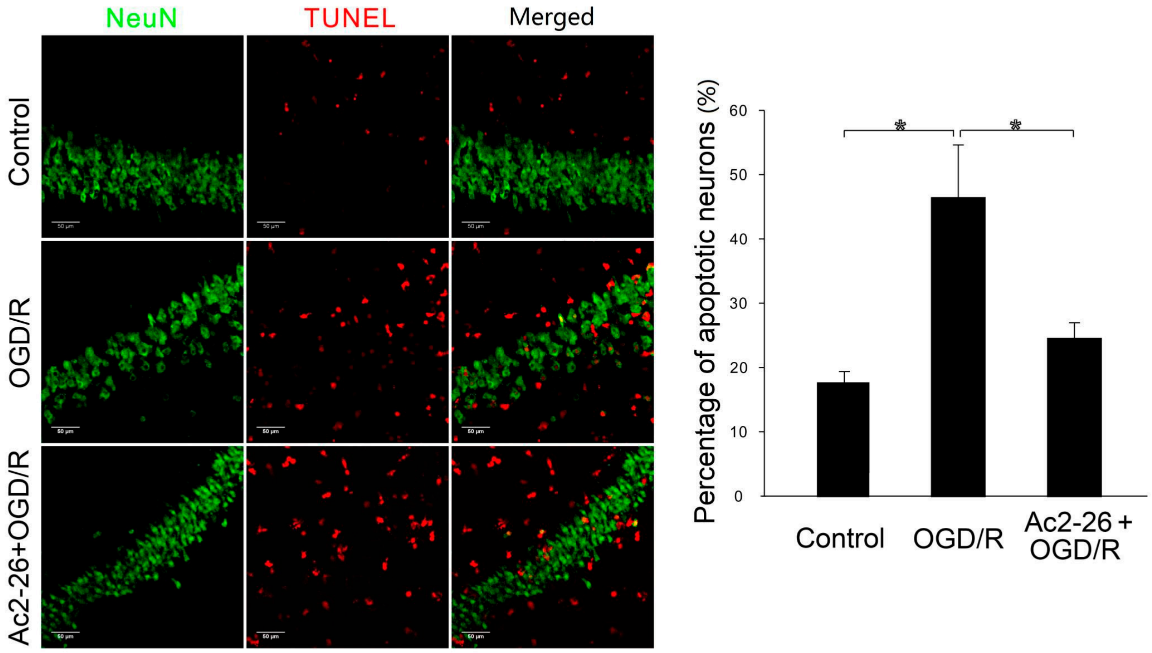

Figure S1. Neural injury was assess by doing TUNEL (TdT-mediated dUTP-biotin Nick End Labeling) assay. The neural injury in CA1 (cornu ammonis 1) region of rat hippocampus was exacerbated after oxygen–glucose deprivation/reperfusion (OGD/R). Formyl peptide receptor (FPRs) activation (Ac2-26 treatment) significantly decreased the neural injury induced by OGD/R. Scale bar: 50 μm; * $p < 0.05$.

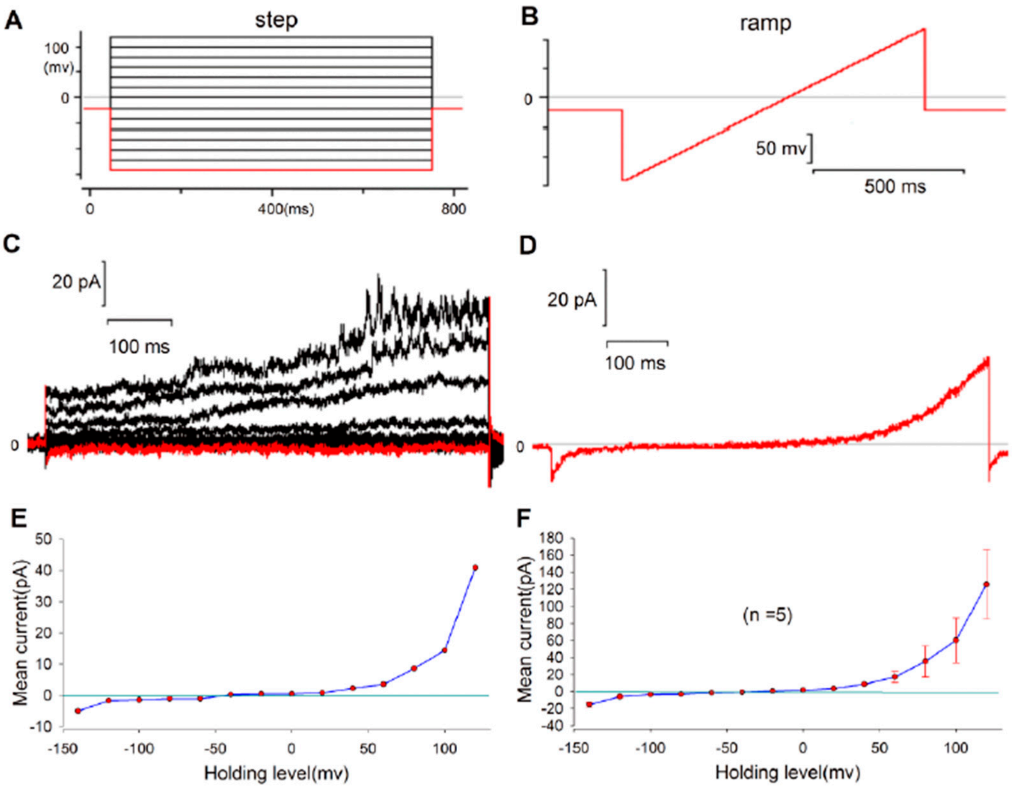

Figure S2. Step voltage and ramp voltage recordings were obtained to characterize membrane currents of BV-2 cells. (**A**,**B**) The stimulus protocol for the step voltage and ramp voltage used in the present experiment; (**C**) The currents of a representative cell recorded using the step voltage procedure; (**D**) Currents of a representative cell (the same cell in (**C**)) recorded using the ramp voltage procedure; (**E**) Current-voltage (I–V) curve for the recordings of the cell in (**C**); (**F**) The statistical results for I–V curves compiled from five individual cells.
